# Introducing Computer-Based Testing in High-Stakes Exams in Higher Education: Results of a Field Experiment

**DOI:** 10.1371/journal.pone.0143616

**Published:** 2015-12-07

**Authors:** Anja J. Boevé, Rob R. Meijer, Casper J. Albers, Yta Beetsma, Roel J. Bosker

**Affiliations:** 1 Department of Psychometrics and Statistics at the Heymans Institute of the University of Groningen, Groningen, the Netherlands; 2 Donald Smits Center for Information Technology, Educational Support and Innovation at the University of Groningen, Groningen, the Netherlands; 3 Department of Educational Science, University of Groningen, Groningen, the Netherlands; University of Leuven, BELGIUM

## Abstract

The introduction of computer-based testing in high-stakes examining in higher education is developing rather slowly due to institutional barriers (the need of extra facilities, ensuring test security) and teacher and student acceptance. From the existing literature it is unclear whether computer-based exams will result in similar results as paper-based exams and whether student acceptance can change as a result of administering computer-based exams. In this study, we compared results from a computer-based and paper-based exam in a sample of psychology students and found no differences in total scores across the two modes. Furthermore, we investigated student acceptance and change in acceptance of computer-based examining. After taking the computer-based exam, fifty percent of the students preferred paper-and-pencil exams over computer-based exams and about a quarter preferred a computer-based exam. We conclude that computer-based exam total scores are similar as paper-based exam scores, but that for the acceptance of high-stakes computer-based exams it is important that students practice and get familiar with this new mode of test administration.

## Introduction

Computer-based exams (CBE) have a number of important advantages compared to traditional paper-based exams (PBE) such as efficiency, immediate scoring and feedback in the case of multiple-choice question exams. Furthermore CBE allow more innovative and authentic assessments due to more advanced technological capacities [1, 2]. Examples are the use of video clips and slide shows to assess medical students in surgery [3] or the use of computer-based case simulations to assess social skills [4]. However, there are also drawbacks when administering CBE such as the additional need for adequate facilities, test-security, back-up procedures in case of technological failure, and time for staff and students to get acquainted with new technology [1].

In order to ensure a smooth transition to computer-based examining in higher education, it is important that students perform equally well on computer-based and paper-based administered exams. If, for example, computer-based administration would result in consistently lower scores than paper-based administration, due to unfamiliarity with the test mode or due to technical problems this would result in biased measurement. Thus, it is important that sources of error, or construct irrelevant variance [5], which may occur as a result of administration mode, are prevented or minimized as much as possible in high-stakes exams. As will be discussed below, however, it is unclear from the existing literature whether the different administration modes will result in similar results.

The adaptation and integration of computer-based testing is developing rather slowly in higher education [6]. Besides institutional and organizational barriers, an important implementation consideration is also the acceptance of CBE by the students [6, 7]. However, as Deutsch et al. [6] discussed “little is known about how attitudes toward computer based assessment change by participating in such an assessment”. Deutsch et al [6] found a positive change in students’ attitudes after a computer-based assessment. As with many studies in prior research (ie. [6, 7]), this took place in the context of a mock exam that was administered on a voluntary basis. There is little research on student attitudes in the context of high-stakes exams, where students do not take the exam on a voluntary basis.

The aim of the present study was to extend the literature on high stakes computer-based exam implementation by (1) comparing student performance on CBE with performance on PBE and (2) evaluating students’ acceptance of computer-based exams. Before we discuss the design of the present study, however, we first discuss prior research on student performance, and acceptance of computer-based multiple-choice exams. The present study is limited to multiple-choice exams as using computer-based exams in combination with open-question or other format tests, may have different advantages or disadvantages, and the aim of this paper was not to study the validity of various response formats.

### Student performance in computer and paper-based tests

The extent to which different administration modes lead to similar performance in educational tests has been investigated for different levels of education. A meta-analysis on test-administration mode in K-12 (primary and secondary education) reading education showed that there was no difference in performance between computer-based and paper-based tests [8]. A meta-analysis on computer-based and paper-based cognitive test performance in the general population (adults) showed that cognitive ability tests were found to be equivalent in different modes, but that there was a difference in performance on speeded cognitive processing tests, in favor of paper-based tests [9]. In the field of higher education, however, as far as we know meta-analyses have not been conducted and results from individual studies seem to vary.

To illustrate the diversity of studies conducted, Table 1 shows some characteristics of a number of studies investigating difference in performance between computer-based and paper-based tests with multiple-choice questions in the context of higher education. The studies vary in the number of multiple-choice questions included in the exam, in the extent to which the exam was high-stakes, and in the extent to which a difference in performance was found in favor of a computer-base or paper-based mode of examining. While our aim was not to conduct a meta-analysis, Table 1 also shows that many studies do not provide enough statistical information to compute an effect-size. Furthermore, not all studies include a randomized design, implying that a difference cannot be causally attributed to mode of examining. Given these varying findings, establishing that administration mode leads to similar performance remains an important issue to investigate.

**Table 1:** Studies investigating performance differences between paper-based and computer-based tests with multiple-choice questions.

	Number of multiple-choice questions	Randomized	High-Stakes	Effect size(Cohen’s d)	Result in favor of
**Lee & Weekaron** [10]	40	**no**	yes	.685	paper-based
**Clariana & Wallace** [11]	100	**yes**	yes^a^	.755	computer-based
**Cagiltay & Ozalp-Yaman** [12]	20	**yes**	yes	.146	computer-based
**Bayazit & Askar** [13]	6	**yes**	unclear	.323	paper-based
**Nikou & Economides** [14]	30	**yes**	unclear	.185	computer-based
**Anakwe** [15]	25	**no**	yes	not possible	
**Frein** [16]	3	**no**	unclear	not possible	
**Rickets & Willks** [17]	unclear	**no**	yes	not possible	
**Kalogeropoulos et al**. [18]	unclear^b^	**yes**	unclear	not possible	

^**a**^ the test counted for 15% of the final grade

^**b**^5 mc-items—but reported means for the mc-test are larger than 5

### Student acceptance of computer-based tests

It is important to understand student acceptance of computer-based testing because the test-taking experience is substantially different from paper-based exams [19]. In paper-based exams with multiple-choice questions, several questions are usually presented per page, and students have the complete exam at their disposal throughout the time allotted to complete the exam. Common test-taking strategies for multiple-choice exams include making notes, marking key words in specific questions, and eliminating answer categories [20, 21]. In computer-based multiple-choice exams however, standard software may not offer these functionalities. For an example where these functionalities were included see McNulty et al. [22]. A study by Hochlehnert et al. [23] in the German higher education context showed that only 37% of students voluntarily chose to take a high-stakes exam via the computer, and that test-taking strategies were a reason why students opted for the paper-based exam. Deutsch et al. [6] showed that the attitudes of medical students in Germany became more positive towards computer-based assessment after taking a practice exam. The context in which students take a mock-exam however, is very different to the actual environment of a formal high-stakes exam. Therefore it is important to investigate both the test-taking experience and student acceptance of computer-based exams in a high-stakes exam.

#### The present study

The present study took place in the last semester of the academic year 2013/2014 with psychology students in the first year of the Bachelor in Psychology program. The university opened an exam facility in 2012 to allow proctored high-stakes exams to be administered via the computer. In the academic year 2012/2013 there were 101 computer-based exams, and this number increased to 225 exams in 2013/2014. Of these exams, 102 were multiple-choice exams, 155 were essay question exams, 58 were a mix of both formats, and 11 exams were in a different format. Most computer-based exams were implemented via the university’s online learning platform NESTOR which is embedded in Blackboard (www.blackboard.com), but has extra programming modules developed by the university. Within the broad project to implement computer-based exams, an additional collaboration of faculties started a pilot project to facilitate computer-based exams through the Questionmark Perception (QMP) software (www.questionmark.com). Of the multiple-choice exams administered over the two-year period, 62 were administered via QMP and 40 were administered via Blackboard. Nevertheless, the program of psychology had no previous experience with computer-based examining.

The psychology program is a face to face based program (in contrast to distance learning). However, for the course that was included in the present study, attending lectures was not mandatory, and students had the option to complete the course based on self-study alone, given that they showed up for the midterm and final exam.

## Methods

To evaluate student performance in different exam modes and acceptance of computer-based exams, computer-based examining was implemented in a Biopsychology course, which is part of the undergraduate psychology program. Assessment of the Biopsychology course consisted of two exams receiving equal weight in grading, and were both high stakes proctored exams. Since the computer-based exam facilities could not facilitate the whole group of students, half of the students were randomly assigned to make the midterm exam by computer, and the other half of the students were assigned to make the final exam by computer. Students did have the opportunity to opt out of computer-based testing and do the exam in the conventional paper/pencil way, and several students made use of this opportunity as will be discussed later. Students were explicitly given the possibility to opt-out of taking a conventional paper-and-pencil exam and take both exams via computer, and no students approached us with this request. Had students approached us with this request however, they would have been granted permission if the capacity of the computer-based exam facilities would have allowed it.

In order to examine whether there were mode differences in student performance on both exams, we analyzed student performance. Student performance data is collected by the University of Groningen for academic purposes. In line with the university’s privacy policy, these data can be used for scientific research when no registered identifiable information will be presented (S1 Policy). Since the analysis of student grades presented in this study entails comparing summary measures of student grades for particular exam mode, no registered identifiable information is presented. Therefore, written informed consent for the use of student grades for scientific research purposes was not obtained.

In order to examine student acceptance of computer-based exams, a questionnaire was placed on the exam desks of students, which they could voluntarily fill out, with the knowledge that their response to the evaluation questionnaire could be used for scientific purposes. Furthermore, students were notified of this procedure at the onset of the course. We did not ask students for written informed consent as to whether they were willing to fill out the questionnaire since they were able to choose to fill out the questionnaire voluntarily and anonymously. Since students were aware that their responses would be used for scientific purposes, informed consent was implied when students chose to fill out the questionnaire. This study, including the procedure for informed consent, was approved by, and adhered to the rules of the Ethical Committee Psychology of the University of Groningen (http://www.rug.nl/research/heymans-institute/organization/ecp/).

In the psychology program, this was the first time a computer-based exam was implemented. The total assessment of the course in biopsychology consisted of a midterm and final exam, which both contributed equally to the final grade, were high-stakes, and took place in a proctored exam hall. At the start of the course students were randomly assigned to make the midterm exam either by computer or as a paper/pencil test. Subsequently the mode of examining was switched for the final exam, so that everyone was assigned to take either the midterm or the final exam as a computer-based test. After completing the computer-based exam, students were invited to fill-out a paper-and-pencil questionnaire on their experience with the computer-based exam, which they could submit before leaving the exam hall. Students received immediate feedback on their performance on the exam in the computer-based condition (number of questions correct), and thus knew their performance on the exam when completing the questionnaire. Students in the paper-based condition received the exam result within a couple of days after taking the exam.

### Participants

At the start of the course, 401 students were enrolled for the course via the digital learning environment, and were randomly assigned to make the midterm exam via paper-based mode or computer-based mode. If a student was assigned to complete the midterm on paper, the final exam would be completed by computer and vice versa. All students who completed a computer-based exam were invited to evaluate their experience by responding to a paper-based questionnaire directly after completing the CBE, with a response rate of 95%. Of those who responded to the questionnaire, 30% was male and 97% aged 18–24 (M = 19.9, SD = 1.34), 3% aged 25 or older. As can be expected in a field experiment, however, there was both some attrition and non-compliance which we will discuss below.

### Attrition and compliance

**Fig 1:**
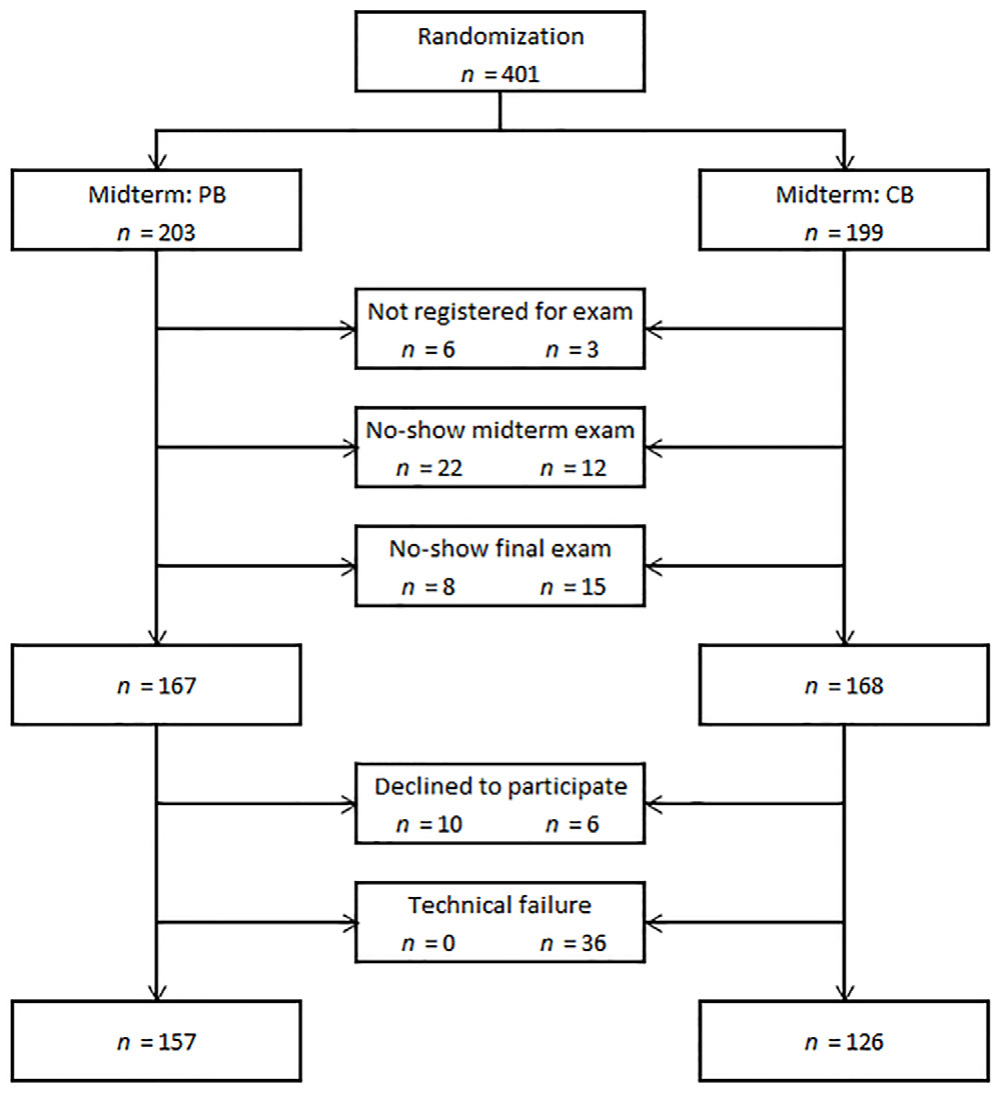
Flow-chart illustrating response from the initial randomization to the actual outcome.

Fig 1 shows the number of eligible participants who were randomly assigned, and the subsequent attrition and non-compliance. There were three sources of attrition: 1) not registering for the exams, 2) registering but not showing up at the midterm, and 3) completing the midterm but not showing up for the final exam. These three sources of attrition led to a 16% overall attrition rate (66 students). Fishers’ exact test (S1 Code) was conducted to investigate whether there was a relationship between random assignment and attrition, with a resulting *p* = 0.06 showing that there was no relationship between random assignment and type of attrition. Therefore, we conclude that it is unlikely that attrition affected the randomization in this field-experiment. There were 16 students who declined taking a computer-based exam at all, and completed both the midterm and final exam on paper. In addition, there was a technical failure at the midterm exam, as a result of which 36 students needed to switch to a paper-based exam in order to be able to complete the exam.

### Materials

#### Student performance

Both the midterm and final exam contained 40 multiple-choice questions with four answer categories. The exams measured knowledge of different topics in biopsychology. The material that was tested on the midterm exam, was not tested again in the final exam. Thus the two exams covered different material included in the course and each exam had an equal weight in determining the final grade. The midterm exam appeared to be somewhat more difficult (mean item proportion correct equal to .70) compared to the final exam (.75). Item-total correlations were somewhat higher for the computer-based exam compared to the paper-based exam (mean 0.32 at both the midterm and final computer-based exam, versus .29 at the midterm paper-based exam and .27 at the final paper-based exam). Reliability estimates showed that the computer-based midterm (α = .78, 95% CI {.72, .83}) and final (α = .75, 95% CI {.67, .80}) exam were slightly more reliable than the paper- based midterm (α = .71, 95% CI {.66, .76}) and final (α = .66, 95% CI {.59, .73}) exam. Student performance in both modes was investigated by comparing the mean number of questions correct on each exam.

#### Acceptance of computer-based tests

Student acceptance was operationalized in three ways (see Table 2). First, students answered questions about their test-taking experience during the computer-based exam and in paper-based exams in general. Second, students were asked whether they preferred a computer-based exam, paper-based exam or did not have a preference. Thirdly, students were asked whether they changed their opinion about computer-based exams as a result of taking a computer-based exam. Answers to the questions on test-taking experience were given on a five-point Likert response scale ranging from ‘completely disagree’ to ‘completely agree’. The question on whether students’ opinions changed had five response options: ‘yes, more positive’, ‘yes, more negative’, ‘no, still positive’, ‘no, still negative’, and ‘no, still indifferent’.

**Table 2:** Evaluations of students test-taking experience and acceptance of computer-based exams.

Student acceptance of computer-based exams	
Questions	Sub-questions
In this computer-based exam	I was able to work in a structured manner
	I had a good overview of my progress in the exam
	I was able to concentrate well
In paper-based exams in general	I am able to work in a structured manner
	I have a good overview of my progress in the exam
	I am able to concentrate well
I prefer a: paper-based exam, computer-based exam, no preference	
Did your opinion about computer-based exams change as a result of taking this exam?	

#### Procedure

The midterm computer-based exam was administered through the Questionmark software, but as mentioned above, there was a technical problem. Since the technical issue could not be solved in time, the final exam was administered directly via Nestor (the university’s online learning platform). As a result of the change in interface, the design and layout of the computer-based midterm and final exam was slightly different. The midterm exam, administered through QMP, was designed so that all questions were presented simultaneously with a scrolling bar for navigation. In the final exam, administered via Nestor, the questions were presented one at a time and navigation through the exam was done via a separate window with question numbers allowing students to review and change answers given to other questions. For both exams, therefore, students had the opportunity to go back and change their answers at any point and as many times as they liked before submitting their final result. After submitting their final answers to both the midterm and final exam in the computer-based mode, students immediately received an indication of how many questions they answered correctly. For the paper-based mode of examining, students took a list of their recorded answers home, and could calculate an indication of how many questions they answered correctly several days after the exam when the answer key was made available in the digital learning environment.

One reviewer suggested that it would be better to use nonparametric statistics to analyze our results because we analyze Likert-scale data. However, parametric statistical approaches are perfectly applicable to Likert scale data [24]. Statistical tests are not based on individual rating scores but on sample means and these means have sampling distributions close to normal. In many cases it is even better to use parametric methods because their base rate power is much higher than nonparametric methods. This is explained in an excellent paper by Norman titled “Likert scales, levels of measurement and the ‘‘laws” of statistics” [24].

## Results

### Student Performance

Table 3 shows that there was no significant difference in the mean-number of questions answered correctly between the computer-based and paper-based mode for both the midterm and final exam.

**Table 3:** Mean number of questions correct in the different exam conditions for the midterm and final exam.

	Computer-based		Paper-based			
	*n*	M(SD)	*n*	M(SD)	*t*(df)	*p*
**midterm exam**	126	28.56 (5.3)	157	28.50 (4.6)	-0.1 (281)	.92
**final exam**	157	29.92 (4.6)	126	29.50 (4.3)	-0.78 (281)	.44

### Student acceptance of CBE

#### Test-taking experience

**Fig 2:**
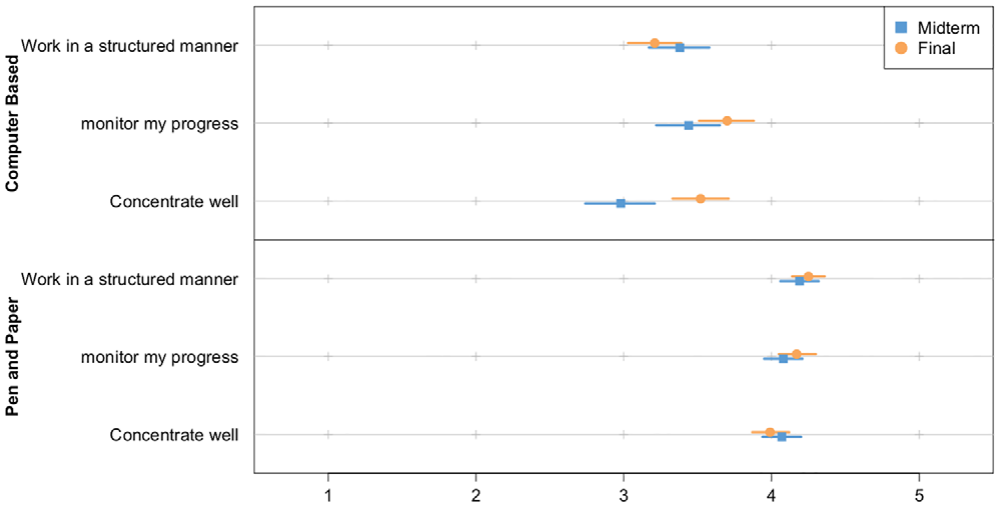
Mean scores and 95% confidence intervals for student approaches to completing the computer-based exam, and paper-based exams in general for the midterm and final exam.

In Fig 2 the mean scores on the questions with respect to test taking experiences for the midterm and final exam are provided. A multivariate ANOVA was conducted to examine whether these questions were evaluated differently for the midterm and final exam. Results of the overall model test (α = .05) showed that there was a difference in how the questions were evaluated between the midterm and final exam (*F*(6,258) = 7.021, *p <* .001, partial-*η*² = .14). Additional (Bonferroni corrected) univariate analyses showed that students were less able to concentrate in the midterm computer-based exam compared to the final exam. (*F*(1,265) = 22.39, *p* = .00014, partial-*η*² = 0.054). See S1 Table for more details on the means, standard-deviations, and effect sizes of this analysis.

To examine the difference in test-taking experience between the computer-based exam and paper-based exams in general, Bonferroni corrected (α = .017) dependent-sample *t*-tests were conducted. Table 4 shows that students are more positive in terms of their ability to work in a structured manner, monitor their progress, and concentrate during paper-based exams compared to the computer-based exam, with medium (0.33) to large (0.64) effect sizes.

**Table 4:** Mean difference between computer-based and paper-based exam evaluation, with dependent-sample t-test results and effect-size.

	CB—PB mode M (SD)	95% CI	*t*(df)	*p*	Cohen’s d
**Structured approach to exam**	-0.9 (1.4)	-1.1, -0.8	-10.7(268)	<.001	0.64
**Monitor progress in exam**	-0.5 (1.5)	-0.7, -0.4	- 6.2 (269)	<.001	0.33
**Concentration during exam**	-0.7 (1.5)	-0.9, -0.6	- 8.1 (269)	<.001	0.46

#### Preference for computer-based exams

Overall, 50% of the students preferred a paper-based exam, 28% preferred a computer-based exam, and 22% indicated that they did not have a preference for one mode over another after completing the computer-based exam. There was no difference in preference for a particular exam-mode between students who completed the midterm and final exam via the computer (Fisher’s exact *p* = 0.97).

With respect to the change of opinion towards computer-based assessment after taking a computer-based exam, 16% remained positive, 43% of students felt more positive, 12% remained negative, 14% felt more negative, and 15% remained indifferent towards computer-based exams. Since there were technical difficulties during the midterm exam, the change in opinion towards computer-based exams may have differed for the midterm and final exam. The category ‘yes, more positive’ was selected by 54% of students at the midterm exam, and 63% of students at the final exam. The Chi-square test over the responses to this question, however, showed that the response patterns between the midterm and final exam did not differ at α = .05 (χ²(4) = 7.1, *p* = 0.13).

## Discussion

### Student performance

In line with recent research [12, 13, 14], we found no difference in the mean number of questions correct between computer- and paper-based tests for both the midterm and final exam. Earlier findings in the field of higher education in favor of paper-based tests [10], and in favor of computer-based tests [11], were not replicated in this study. Based on these findings, we can conclude that recent findings show that exam-mode may not cause differential student performance in higher education. An important explanation for this finding could be the population of students in this study. Students in this study entered the higher education system largely directly after completing secondary education and represent a generation that has grown up with technology. Earlier studies on the use of computer-based testing may have found a difference in favor of paper-based tests as a result of test takers’ unfamiliarity with technology. Therefore, the lack of a difference in performance between modes in the present study may be the result of a generational difference in student population compared to older studies. This also implies that current studies with older populations of students may still find a mode effect, although adults today will have had more technology exposure in daily life than studies conducted with adults twenty years ago.

### Student acceptance of CBE

#### Test-taking experience

Students generally indicated that the test-taking experience in PBE in general was more favorable compared to CBE in terms of their ability to work in a structured manner, have a good overview of their progress through the exam, and their ability to concentrate. While there was no difference in performance for computer-based and paper-based exams, these findings suggest that students appear to feel less in control when taking a computer-based exam relative to a paper-based exam. This is in line with previous findings by Hochlehnert et al. [23] who found that the absence of functionality to apply test-taking strategies was a reason for students not to choose a computer-based exam. Further research is necessary to see if this difference in approach to taking the exam may be an artefact of the first-time introduction to computer-based exams. Students who regularly take computer-based exams may be more accustomed to this mode, and therefore have developed confidence in their approach to taking computer-based exams. Another avenue that may be pursued in order to better understand the test-taking experience in CBE may be to extend the research of Noyes, Garland and Robbins [25] who found that students experienced a higher cognitive load in a short computer-based multiple-choice test compared to an equivalent paper-based test. Further research could investigate the extent to which the perceived test-taking experience is related to cognitive load.

We found that students who took the final exam by computer, were able to concentrate better on average than students who took the midterm exam by computer. The first possible explanation for this result, may be the technical problem during the midterm. Students in the computer-based exam hall who did not experience the technical problem, may have been affected indirectly by the unrest in the exam hall as the directly affected students were provided with a paper-based exam. If this were the explanation for the difference in concentration between the midterm and final exam, it would seem logical that students who completed the midterm exam were also more negative about computer-based exams compared to the group of students who completed the final exam by computer. We found no difference however, in the extent to which student opinions became more negative towards CBE after taking the computer-based exam.

Another possible explanation for the difference in the ability to concentrate between the midterm and final exam is the design of the computer-based assessment. A difference in design was noted by Rickets and Wilks [17] to explain improved student performance in CBE when the design was changed from scrolling to a one-question-at-a-time presentation design. In the present study all the questions were displayed simultaneously in the midterm file, while in the final exam questions were presented one at a time. In presenting questions one at a time during the final exam students may have been able to focus better on the questions at hand, explaining the greater ability to concentrate reported by students.

#### Preference for CBE

Approximately 50% of the students indicated a preference for paper-based multiple-choice exams after taking their first computer-based exam. About 25% indicated no preference, and another 25% indicated a preference for computer-based assessment. Interesting is why students prefer a particular exam mode, and whether the experience of taking a computer-based exam can make a difference for the acceptance of computer-based exams.

Earlier research by Hochlehnert et al. [23] found that given a choice, 37% of students chose to complete a high-stakes CBE, and Deutsch et al. [6] found that about 65% of the students were prepared to take a (low-stakes) computer-based mock exam. Furthermore, Deutsch et al. [6] found that of the students who participated, 36% of students were more positive, 20% were more negative, and 44% did not change their opinion about CBE after taking a computer-based mock-exam. To compare, in the present study, 43% of the students were more positive, 14% more negative, and 43% did not change their opinion towards CBE as a result of taking the computer-based exam. While the present study used a somewhat different operationalization than the Deutsch et al. [6] study, it is clear that overall student acceptance can improve with more experience with computer-based testing.

Another reason why students may have become more positive in their opinion about computer based testing is that they received immediate feedback on their exam performance. This could be particularly relevant for students completing the final exam by computer, since receiving the result immediately would allow students to calculate whether they passed the course as a whole, while students in the midterm would not have had this opportunity since both exams need to have been completed in order to determine whether the course was passed. Nevertheless, more research is needed to understand why some students remain negative, or become more negative towards CBE after taking a computer-based test.

### Practical implications

Based on the above discussion there are several practical implications for Universities seeking to implement CBE. Student performance on multiple choice question exams does not appear to vary across test mode. The benefits of CBE, and the lack of negative consequences, can both be used in the communication towards students prior to the first implementation of CBE in order to maximize acceptance. Furthermore, universities need to invest in good CBE exam facilities. This includes investing in adding more test-taking functionalities so that students test-taking experience may be as optimal as possible. Furthermore, the potential of technical failure is a risk that requires good protocols so that students are able to complete the exam either on a different computer or on paper.

The full potential of computer-based tests can be realized in further developments. One option is to use computer adaptive testing (CAT). The advantage of CAT is that items are chosen from an item pool that best fit the level of the candidate. In many higher education institutes however, this is difficult to realize as a very large item pool with regular refreshment is needed. In combination with the extensive psychometric knowledge necessary for this development, this is generally beyond the scope of many university courses. What may be easier to realize however, is to offer test items to students in random order, which helps prevent cheating.

### Limitations

There were several limitations to the present study. First, there were technical problems during the midterm computer-based exam. As a result of this technical failure a number of students had to complete the planned CBE on paper. This remains a risk for computer-based exams in general, and the facilities for computer-based examining need to be organized in such a way that when this occurs unexpectedly in practice, hindrance for students is minimized. In the present study, students were allowed extra time to complete the exam, although no one made use of it. It is important to note, that while students may not have a good test-taking experience, their results are unlikely to suffer as a consequence. Several studies have shown student performance in CBE is not affected by technical issues [26, 27].

An important aspect of introducing computer-based assessment deserves mention as well, namely the teacher of faculty perspective. Since the present study was conducted in a single course, the teacher perspective was outside the scope of the present study. Research into teacher acceptance and willingness to implement computer-based assessment may also provide relevant insight into improving the implementation of computer-based exams in higher education.

Furthermore, our sample consisted of students who were primarily ‘traditional’ students and started their study soon after completing high school. A population containing more mature aged students may view technology differently. In addition students were studying face to face. Students who study via distance mode may view computer-based testing differently than face-to-face students.

## Conclusion

This study found that students performed equally well in computer-based multiple-choice exams compared to paper-based exams. While paper-based exams may be the norm in many universities, investing in computer-based exams may be beneficial for the younger generation who are more and more growing up with computer and digital technologies. Further research is necessary into the optimal design of computer-based exams, such that student-acceptance is maximized and not an irrelevant source of stress during exams in a high-stakes context.

## Supporting Information

S1 Code (DOCX)

S1 Data (SAV)

S2 Data (SAV)

S1 Policy (PDF)

S1 Table (DOCX)
