# Supplementary material for: Introducing Computer-Based Testing in High-Stakes Exams in Higher Education: Results of a Field Experiment
Source: PLoS One. 2015 Dec 7;10(12):e0143616. doi: 10.1371/journal.pone.0143616 (PMC4671535; doi:10.1371/journal.pone.0143616)
Supplement: S1 Code — (DOCX) [file pone.0143616.s001.docx]

**S1 Code**

#Matrix with the number of dropouts per assigned condition

att<- matrix(c(3,12,15,6,22,8),nrow=3,ncol=2)

#Fisher exact test

tes<- fisher.test(att, simulate.p.value=TRUE)
