# Supplementary material for: Introducing Computer-Based Testing in High-Stakes Exams in Higher Education: Results of a Field Experiment
Source: PLoS One. 2015 Dec 7;10(12):e0143616. doi: 10.1371/journal.pone.0143616 (PMC4671535; doi:10.1371/journal.pone.0143616)
Supplement: S1 Policy — (PDF) [file pone.0143616.s004.pdf]

REGELING BESCHERMING PERSOONSGEGEVENS STUDENTEN EN PERSONEEL

I Begripsbepalingen

Artikel 1

1. In deze regeling wordt verstaan onder:
   a. **Persoonsgegeven:** elk gegeven betreffende een geïdentificeerde of identificeerbare betrokkene;
   b. **verwerking van persoonsgegevens:** elke handeling of elk geheel van handelingen met betrekking tot persoonsgegevens;
   c. **betrokkene:** degene op wie een persoonsgegeven betrekking heeft;
   d. **verantwoordelijke:** het College van Bestuur dat, alleen of tezamen met anderen, het doel van en de middelen voor de verwerking van persoonsgegevens vaststelt;
   e. **beheerder:** de functionaris die door de verantwoordelijke aangewezen is om in het kader van deze namens de verantwoordelijke bepaalde taken en bevoegdheden uit te oefenen;
   f. **sub-beheerder:** de functionaris, belast met de feitelijke administratie van een categorie van persoonsgegevens, die door de beheerder is aangewezen namens hem bepaalde taken en bevoegdheden uit te oefenen;
   g. **bewerker:** degene die ten behoeve van de verantwoordelijke persoonsgegevens verwerkt, zonder aan zijn rechtstreeks gezag te zijn onderworpen;
   h. **verstrekken van persoonsgegevens:** het bekend maken of ter beschikking stellen van persoonsgegevens;
   i. **toestemming van de betrokkene:** een vrije, specifieke en op informatie berustende wilsuiting waarmee de geregistreerde aanvaardt dat hem betreffende gegevens worden verwerkt;
   j. **student:** natuurlijk persoon, ingeschreven bij de Rijksuniversiteit Groningen als student, extraneus of cursist, alsmede diegene die aan de Rijksuniversiteit Groningen heeft gestudeerd;
   k. **personeel:** natuurlijk persoon die aangesteld is (geweest) bij de Rijksuniversiteit Groningen alsmede diegene die zonder dat er sprake is van een aanstelling werkzaamheden verricht(te) in universitair verband.

II Beheer van gegevens

Artikel 2

Het College van Bestuur is de verantwoordelijke voor de verwerking van persoonsgegevens.

Artikel 3

1. Het Hoofd van de University Student Desk (USD) is beheerder van de categorieën van gegevens betreffende studenten die berusten bij de centrale studentenadministratie en bij de centraal-universitaire studentenvoorzieningen.
2. Het Hoofd van de afdeling Personeel en Organisatie (P&O) van de RUG is beheerder van de categorieën van persoonsgegevens betreffende personeel die berusten op centraal niveau.
3. De faculteitsbestuurders en directies van diensten zijn beheerders van de categorieën van gegevens die daar berusten.
4. De beheerders kunnen de taken en bevoegdheden als genoemd in artikel 8 leden 1, 4 en 5 opdragen aan een sub-beheerder.
5. Een ieder die handelt onder het gezag van de verantwoordelijke of van de beheerder, alsmede de beheerder zelf, voor zover deze toegang hebben tot persoonsgegevens, verwerkt deze slechts in opdracht van de verantwoordelijke, behoudens afwijkende wettelijke verplichtingen.
6. Personen, bedoeld in lid 4, zijn verplicht tot geheimhouding van de persoonsgegevens waarvan zij kennis nemen, behoudens voor zover enig wettelijk voorschrift hen tot mededeling verplicht of uit hun taak de noodzaak tot mededeling voortvloeit.

III Verzameling en verwerking van gegevens

Artikel 4

De gegevens betreffende studenten worden verzameld ten behoeve van:

a. administratieve handelingen met betrekking tot inschrijving en tot het berekenen, vastleggen en innen van college- en examengelden;
b. de voorbereiding van beleidsbeslissingen op het gebied van het onderwijs;
c. het samenstellen van de kiesregisters;
d. de werkzaamheden van de universitaire instanties die zich richten op het studentenwelzijn;
e. het nemen van beslissingen met betrekking tot de inrichting van het onderwijs;
f. het vastleggen van tentamenuitslagen;
g. het vaststellen van examenresultaten;
h. de studieadvisering en – ondersteuning;
i. het verzenden van voor studenten relevante informatie;
j. de beveiliging van de universitaire gebouwen.

Artikel 5

De gegevens betreffende personeel worden verzameld ten behoeve van:

a. het uitoefenen van de personeelsbeheerfunctie en de daarvoor noodzakelijke administratieve handelingen;
b. de voorbereiding van de door de bestuursorganen van de RUG te nemen beleidsbeslissingen;
c. het samenstellen van de kiesregisters;
d. de dienstverlening aan het personeel en ten behoeve van werkzaamheden van aan de universiteit verbonden instanties die zich richten op het welzijn van het personeel;
e. de personeelsbegeleiding en beoordeling;
f. het verzenden van voor het personeel relevante informatie;
g. de beveiliging van de universitaire gebouwen.

Artikel 6

1. De gegevens mogen alleen worden verwerkt voor zover dit:
   a. niet onverenigbaar is met de doelstellingen van de gegevensverzamelingen genoemd in de artikelen 4 en 5 en in overeenstemming is met de overige bepalingen van deze regeling, of;
   b. geschiedt met uitdrukkelijke toestemming van betrokkene, of;
   c. verplicht is op grond van een wettelijk voorschrift.
2. Bij de beoordeling of een verwerking verenigbaar is met de doelstelling waarvoor ze verkregen zijn wordt in elk geval rekening gehouden met:
   a. de verwantschap tussen het doel van de beoogde verwerking en het doel waarvoor de gegevens zijn verkregen;
   b. de aard van de betreffende gegevens;
   c. de gevolgen van de beoogde verwerking voor betrokkene;
   d. de wijze waarop de gegevens zijn verkregen;
   e. de mate waarin jegens betrokkene wordt voorzien in passende waarborgen.
3. Verdere verwerking van gegevens voor historische, statistische of wetenschappelijke doeleinden wordt niet als onverenigbaar beschouwd, indien de verantwoordelijke de nodige voorzieningen heeft getroffen ten einde te verzekeren dat de verdere verwerking uitsluitend geschiedt ten behoeve van deze specifieke doeleinden.

Artikel 7

1. De verantwoordelijke houdt een register bij van de gegevensverwerkingen.
2. Het register bevat ten minste:
   a. de naam en het adres van de verantwoordelijke;
   b. het doel of de doeleinden van de verwerking;
   c. een beschrijving van de categorieën van betrokkenen en van de gegevens of categorieën van gegevens die daarop betrekking hebben.

IV Verstrekking van gegevens

Artikel 8

1. De beheerder verstrekt uitsluitend persoonsgegevens aan personen en instanties binnen de universitaire gemeenschap, indien dit niet onverenigbaar is met de doelstellingen van de gegevensverwerking.
2. Het College van Bestuur verstrekt uitsluitend persoonsgegevens aan personen en instanties buiten de universitaire gemeenschap, indien dit niet onverenigbaar is met de doelstellingen van de gegevensverwerking.
3. Aan personen en instellingen worden persoonsgegevens beschikbaar gesteld, indien daartoe een wettelijke verplichting bestaat.
4. Persoonsgegevens kunnen alleen dan zonder toestemming van de betrokkene worden verstrekt ten behoeve van wetenschappelijk onderzoek, indien aan alle van de volgende voorwaarden is voldaan:
   a. het vragen van de gerichte toestemming is in redelijkheid niet mogelijk;
   b. het onderzoek dient een algemeen belang;
   c. het onderzoek kan niet zonder de desbetreffende gegevens worden uitgevoerd;
   d. de persoonlijke levenssfeer van de betrokkene wordt niet onevenredig geschaad door de gegevensverstrekking en het staat vast dat het onderzoek niet in de vorm van tot de geregistreerde herleidbare gegevens zal worden gepresenteerd;
   e. het onderzoek wordt verricht conform een op de onderzoeker betrekking hebbende gedragscode.
5. Het College van Bestuur kan aan het Centraal Bureau voor de Statistiek en het Centraal Register Inschrijvingen Hoger Onderwijs die persoonsgegevens van studenten verstrekken die dit bureau nodig heeft voor het samenstellen van statistieken in verband met het wetenschappelijk onderwijs.

6. Door de beheerder van de betreffende categorie van persoonsgegevens van studenten kan van het met goed gevolg afleggen van een afsluitend examen in de Geneeskunde, Tandheelkunde of Farmacie en van het met goed gevolg afleggen van een arts-, tandarts- of apothekersexamen mededeling worden gedaan aan de Geneeskundige Hoofdinspectie.
7. Door de beheerder van de betreffende categorie van persoonsgegevens van studenten kan van het met goed gevolg afleggen van een afsluitend examen en voor wat betreft de opleidingen Geneeskunde, Tandheelkunde of Farmacie tevens van het met goed gevolg afleggen van een arts-, tandarts- of apothekersexamen eenmalig mededeling worden gedaan aan de op het desbetreffende vakgebied werkzame beroepsorganisatie.
8. Indien persoonsgegevens van studenten of personeel zodanig zijn geanonimiseerd dat zij redelijkerwijs niet tot de individuele persoon herleidbaar zijn, kan de verantwoordelijke beslissen deze te verstrekken ten behoeve van wetenschappelijk onderzoek en statistieken.

V Kennisgeving

Artikel 9

1. De verantwoordelijke zal door middel van een algemene kennisgeving het bestaan van de persoonsverwerking en van deze regeling vermelden, alsmede daarin aangeven op welke wijze deze kan worden ingezien en nadere informatie ter zake kan worden ingewonnen.
2. De verantwoordelijke heeft de plicht de betrokkene vooraf gericht te informeren omtrent de aard van deze gegevens die over zijn persoon in de gegevensverwerking worden opgenomen, alsmede omtrent de doeleinden die daarmee worden nagestreefd, een en ander met inachtneming van het bepaalde in artikel 6.

VI Beveiliging en bewaring van gegevens

Artikel 10

1. Het College van Bestuur draagt zorg voor de nodige voorzieningen van technische en organisatorische aard ter beveiliging van de persoonsgegevens tegen onbevoegde verwerking daarvan.
2. Iedere beheerder treft de nodige maatregelen teneinde onbevoegde verwerking van de door hem beheerde gegevens te voorkomen.

Artikel 11

1. Persoonsgegevens worden niet langer bewaard dan noodzakelijk voor de verwezenlijking van de doeleinden waarvoor zij worden verzameld en verwerkt.
2. Persoonsgegevens worden langer bewaard dan bepaald in het eerste lid, indien dit noodzakelijk is voor historische, statistische of wetenschappelijke doeleinden, dan wel op grond van een wettelijk voorschrift, in welk geval zij in een afzonderlijke registratie worden bewaard.

VII Recht van inzage, afschrift en verbetering

Artikel 12

1. Iedere betrokkene heeft recht op inzage in de over hem opgenomen persoonsgegevens. Een verzoek hiertoe wordt schriftelijk ingediend bij de desbetreffende beheerder. Indien de beheerder niet bekend is, wordt het verzoek ingediend bij het College van Bestuur.
2. Op een verzoek om inzage wordt uiterlijk binnen vier weken na indiening schriftelijk gereageerd.
3. Elke betrokkene heeft op verzoek ten hoogste twee keer per jaar recht op een afschrift van de over hem opgenomen persoonsgegevens. De daaraan verbonden kosten kunnen -tot een maximum van 4 euro per verzoek- aan de verzoeker in rekening worden gebracht.
4. Elke betrokkene kan een schriftelijk verzoek tot verbetering van de met betrekking tot hem opgenomen persoonsgegevens indienen bij de beheerder. Indien de beheerder niet bekend is, wordt het verzoek ingediend bij het College van Bestuur.
5. De beheerder verbetert de gegevens indien zij feitelijk onjuist zijn, onvolledig of niet ter zake dienend zijn voor het doel waarvoor ze verwerkt zijn, of op een andere wijze in strijd zijn met deze regeling of met enig wettelijk voorschrift. Hiervan wordt de betrokkene binnen vier weken na indiening van het verzoek schriftelijk in kennis gesteld, alsmede diegenen aan wie onjuiste gegevens van betrokkene zijn verstrekt.
6. Indien de beheerder de verzochte verbetering niet aanbrengt, doet hij hiervan binnen vier weken na indiening van het verzoek gemotiveerd en schriftelijk mededeling aan betrokkene.

VIII Bezwaren

Artikel 13

Tegen een besluit, zoals bedoeld in artikel 1:3 van de Algemene wet bestuursrecht, genomen op grond van deze regeling door of namens het College van Bestuur kan binnen zes weken schriftelijk bezwaar worden gemaakt bij het College van Bestuur:

a. door studenten bij het Centraal Loket Rechtsbescherming Studenten via www.rug.nl/studenten of Postbus 72, 9700 AB Groningen;
b. door personeelsleden via Postbus 72, 9700 AB Groningen.

IX Klachten

Artikel 14

1. Indien de betrokkene van mening is dat de bepalingen van deze regeling niet worden nageleefd en hij hierdoor in zijn belang wordt getroffen, kan hij hierover een klacht indienen:
   a. door studenten bij het Centraal Loket Rechtsbescherming Studenten via www.rug.nl/studenten of Postbus 72, 9700 AB Groningen;
   b. door personeelsleden bij het College van Bestuur via Postbus 72, 9700 AB Groningen.
2. De bepalingen van de Regeling Centraal Loket Rechtsbescherming Studenten, de Algemene Klachtenregeling Rijksuniversiteit Groningen en hoofdstuk 9 van de Algemene wet bestuursrecht zijn van toepassing.

X Slotbepalingen

Artikel 15
In gevallen waarin deze regeling niet voorziet beslist het College van Bestuur.

Artikel 16
Deze regeling wordt aangehaald als:
Regeling Bescherming Persoonsgegevens Studenten en Personeel Rijksuniversiteit Groningen.

Vastgesteld door het College van Bestuur op 27 augustus 2002,
laatstelijk gewijzigd op 27 juli 2010.
