# Supplementary material for: Introducing Computer-Based Testing in High-Stakes Exams in Higher Education: Results of a Field Experiment
Source: PLoS One. 2015 Dec 7;10(12):e0143616. doi: 10.1371/journal.pone.0143616 (PMC4671535; doi:10.1371/journal.pone.0143616)
Supplement: S1 Table — (DOCX) [file pone.0143616.s005.docx]

**S1 Table**

**The approach to taking computer-based exams and paper-based exams in general**

|  | **Midterm exam**  **M (SD)** | **Final exam M(SD)** | ***F*(1, 265)** | ***p-*value** | **Partial η²** |
| --- | --- | --- | --- | --- | --- |
| **In this computer-based exam I was able to:** |  |  |  |  |  |
| a. Work in a structured manner | 3.4 (1.2) | 3.2 (1.1) | 0.71 | .40 | .003 |
| b. Monitor my progress | 3.4 (1.2) | 3.7 (1.1) | 4.52 | .03 | .017 |
| c. Concentrate well | 3.0 (1.3) | 3.5 (1.1) | 14.94 | <.001 | .054 |
| **In paper-based exams in general I am able to:** |  |  |  |  |  |
| a. Work in a structured manner | 4.2 (0.7) | 4.3 (0.7) | 0.59 | .44 | .002 |
| b. Monitor my progress | 4.1 (0.7) | 4.2 (0.8) | 1.36 | .35 | .005 |
| c. Concentrate well | 4.1 (0.7) | 4.0 (0.8) | 0.34 | .56 | .001 |
